# Acyclic nucleoside phosphonates containing the amide bond: hydroxy derivatives

**DOI:** 10.1007/s00706-019-2351-y

**Published:** 2019-03-01

**Authors:** Iwona E. Głowacka, Dorota G. Piotrowska, Graciela Andrei, Dominique Schols, Robert Snoeck, Andrzej E. Wróblewski

**Affiliations:** 1 Bioorganic Chemistry Laboratory, Faculty of Pharmacy, Medical University of Lodz, Muszyńskiego 1, 90-151 Lodz, Poland; 2 Rega Institute for Medical Research, KU Leuven, Herestraat 49, 3000 Louvain, Belgium

**Keywords:** Nucleotides, Amide bond formation, NMR spectroscopy, Phosphonates

## Abstract

**Abstract:**

To study the influence of a linker rigidity and changes in donor–acceptor properties, three series of nucleotide analogs containing a P–X–HN–C(O)– residue (X=CH(OH)CH_2_, CH(OH)CH_2_CH_2_, CH_2_CH(OH)CH_2_) as a replacement for the P–CH_2_–O–CHR– fragment in acyclic nucleoside phosphonates, e.g., adefovir, cidofovir, were synthesized. EDC proved to provide good yields of the analogs from the respective ω-amino-1- or -2-hydroxyalkylphosphonates and nucleobase-derived acetic acids. New phosphorus–nucleobase linkers are characterized by two fragments of the restricted rotation within amide bonds and in four-atom units (P–CH(OH)–CH_2_–N, P–CH(OH)–CH_2_–C and P–CH_2_–CH(OH)–C) in which antiperiplanar disposition of P and N/C atoms was deduced from ^1^H and ^13^C NMR spectral data. The synthesized analogs P–X–HNC(O)–CH_2_B [X=CH(OH)CH_2_, CH(OH)CH_2_CH_2_, CH_2_CH(OH)CH_2_] appeared inactive in antiviral assays on a wide variety of DNA and RNA viruses at concentrations up to 100 μM, while two phosphonates showed cytostatic activity towards myeloid leukemia (K-562) and multiple myeloma cells (MM.1S) with IC_50_ of 28.8 and 40.7 μM, respectively.

**Graphical abstract:**

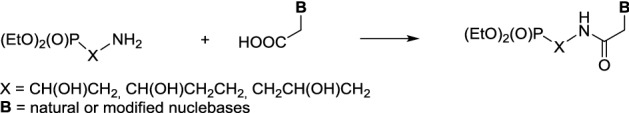

## Introduction

Despite numerous efforts, highly effective antiviral drugs without side effects are not yet available. A search for such compounds appeared even more difficult, since various viruses can undergo fast mutations. Several antiviral medications are at physicians disposal and among them acyclic nucleoside phosphonates (ANPs) belong to the most important [1–3].

**Fig. 1:**
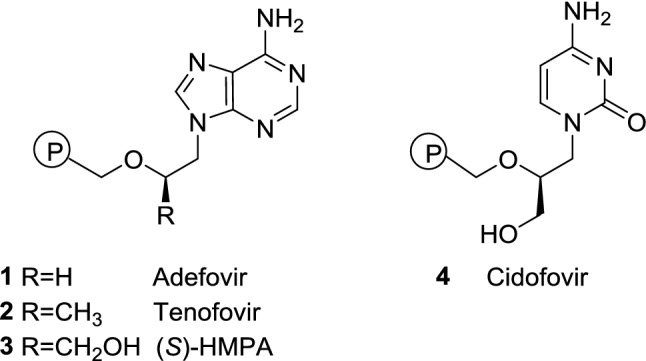
Structures of adefovir (**1**), tenofovir (**2**), (*S*)-HMPA (**3**), and cidofovir (**4**)

Formulations delivering adefovir (**1**), tenofovir (**2**), (*S*)-HMPA (**3**), and cidofovir (**4**) (Fig. 1) have been used for several decades and their structural frameworks stimulated chemists to create modifications that are more active. Linkers connecting nucleobase and phosphonic acid moieties in **1**–**4** seem to be the first choice for modifications but this approach so far did not lead to discovery of more active compounds.

Based on the commonly accepted mechanism of action of ANPs [3, 4], a structure of new analogs has to contain at both termini a P(O)–CH_2_ fragment which is responsible for the stability towards phosphate-cleaving enzymes and nucleobases or their close analogs which facilitate interactions with nucleobases of viral nucleic acids. Preferably, a four-atom linker capable of hydrogen bonding should interconnect these units.

**Fig. 2:**
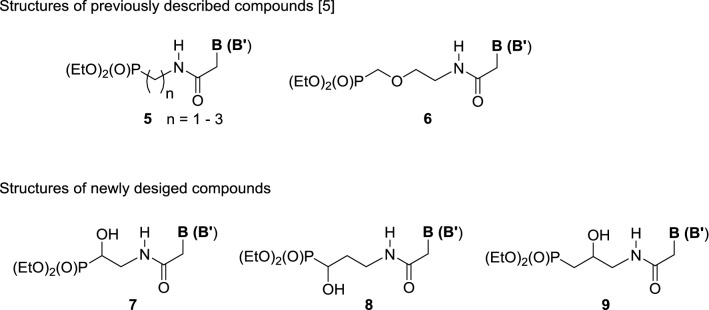
Structures of previously described and newly designed phosphonates as acyclic nucleoside phosphonates (B = canonical nucleobases; B′ = nucleobase analogs: see Scheme 1)

Recently, we put forward an idea of replacing a P–CH_2_–**O**–CHR– fragment in **1**–**4** with the amide bond [5]. This was inspired by the successful application of the isosteric replacement of this bond by a methylene ether [–CH_2_–O] moiety in studies on the biological activity of natural peptides [6–8]. However, the introduction of the amide [P–CH_2_–**HN**–**C**(**O**)–] residue significantly increases donor–acceptor interactions within a new linker. It also modifies its conformational flexibility due to the restricted rotation around the amide bond. In our first approach [5], four series of the phosphonate amides of general formulae **5** and **6** were synthesized (Fig. 2).

In this paper, we continue our efforts to identify structural features in acyclic nucleoside phosphonates containing the amide bond **7**–**9** responsible for their antiviral activity. The phosphonates **5** and **6** containing the aliphatic linkages X (CH_2_, CH_2_CH_2_, and CH_2_CH_2_CH_2_) or ethereal interconnection (CH_2_OCH_2_CH_2_) including the methylene group which secured the separation of nitrogen atoms (N1 or N9) in nucleobases and the phosphorus atom by four bonds and resulted in compounds structurally closest to the drugs **1**–**4** that appeared inactive. Hence, we reasoned that installation of additional polar functionalities in the aliphatic linker could improve acyclic nucleoside–enzyme interactions, and thus significantly increase the activity. Herein, we wish to describe our studies on the synthesis and the biological activity of the new amides **7**–**9** having the hydroxy groups within the aliphatic linker. To synthesize the final acyclic nucleoside phosphonates **7**–**9,** the approach involving the formation of the amide bond between the respective acetic acid derivatives **13a**–**13h** and ω-aminophosphonates **10**–**12** was followed (Scheme 1) [5].

**Figure:**
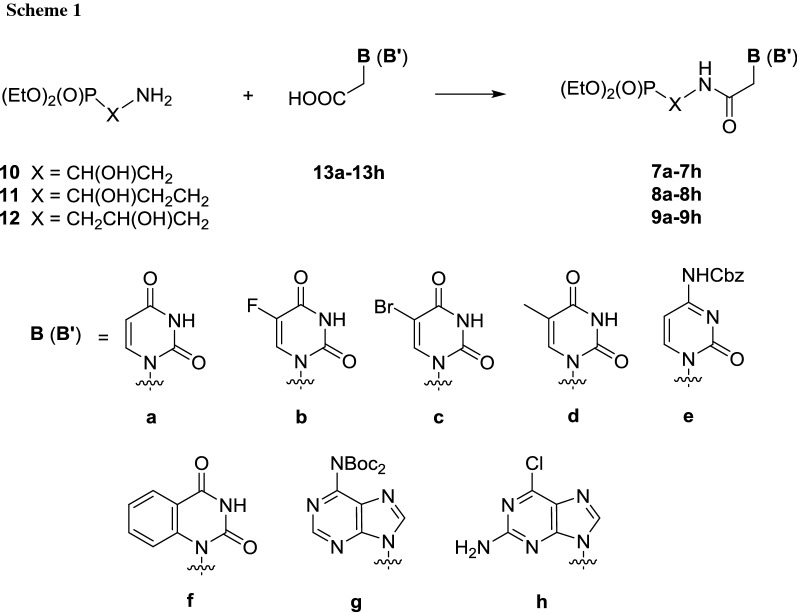

For many years, the biological activity of phosphonate nucleoside analogs has been studied employing free phosphonic acids but recently, they are administered in the form of prodrugs (esters or amides) to significantly improve bioavailability of very polar acids [2, 3, 9–12]. We opted to test phosphonate diethyl esters **7**–**9,** since they to some extent resemble the lipophilic prodrugs in ability to permeable cell membranes. Our recent experience is in line with this strategy as we discovered examples of the biologically active phosphonate diethyl esters substituted with various heterocyclic motives, while the respective free acids appeared inactive [13, 14].

Furthermore, we were afraid of possible dehydration of our hydroxyalkylphosphonates under harsh conditions associated with the application of, for example, acids, bases or even iodotrimethylsilane.

## Results and discussion

To accomplish the synthesis of the second series of the amides **7a**–**7h** to **9a**–**9h**, pure ω-aminophosphonates **10**–**12** have to be efficiently prepared (Schemes 2 and 3). The phosphonate **10** is a known compound [15, 16] and it was prepared by the ammonolysis of diethyl 1,2-epoxyethanephosphonate **14,** but the authors failed to provide a full characterization of the material they obtained. The phosphonate **12** is also known [15] and it was obtained in a similar manner from diethyl 2,3-epoxypropanephosphonate **15**, though the authors were unable to prove the purity of the product. Our experience with the 2,3-epoxypropanephosphonate framework [17] assured us that phosphonates **10** and **12** of the highest purity could be obtained from the epoxides **14** and **15**, respectively, when dibenzylamine will be applied instead of ammonia followed by hydrogenolysis (Scheme 2) [18].

**Figure:**
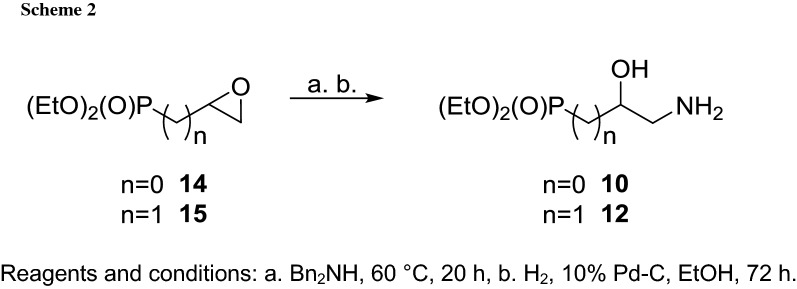

**Figure:**
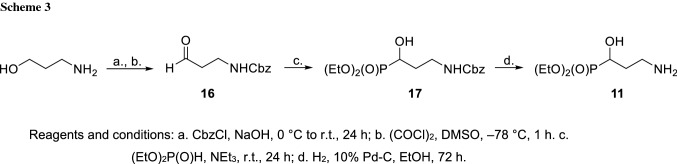

Available synthetic strategies to the phosphonate **11** take advantage of the addition of diethyl phosphite derivatives to N-protected 3-aminopropanal (Scheme 3) [19, 20].

In our hands, N-Cbz protection of 3-aminopropan-1-ol followed by the Swern oxidation proved optimal for the preparation of the aldehyde **16** which was later subjected to a triethylamine-catalyzed phosphorylation to provide a protected hydroxyphosphonate **17**. Final hydrogenolysis gave pure phosphonate **11** in 25% overall yield.

**Fig. 3:**
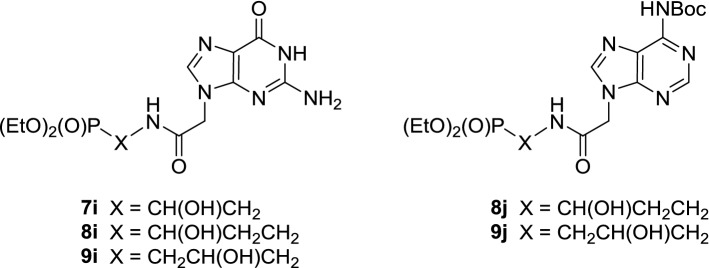
Guanine phosphonate analogs **7i**, **8i**, **9i** and NH-Boc adenine phosphonates **8j** and **9j**

The known uracil- **13a**, 5-fluorouracil- **13b**, 5-bromouracil- **13c**, thymine- **13d** and benzouracil-containing **13f** acetic acids were prepared by alkylation of the respective nucleobases with chloro- or bromoacetic acid [21–25]. Synthesis of amides **7e**, **8e**, **9e** and **7g**, **8g**, **9g** containing cytosine- and adenine-acetyl subunits required prior protection of the starting acids as N-Cbz- and N-diBoc-derivatives **13e** [26] and **13g** [27] to significantly increase lipophilicity of the final products. Following the literature precedences [28] later confirmed by our experience [5], we anticipated to prepare guanine analogs **7i**–**9i** (Fig. 3) from the 2-amino-6-chloropurine precursors **7h**–**9h** (Scheme 1) using 2-amino-6-chloropurin-9-yl)acetic acid **13h** [29] as a substrate.

With the ω-aminophosphonates **10**–**12** of high purity secured, we turned to the coupling with a series of nucleobase-acetic acids (Scheme 1). As established earlier [5], all syntheses of amides **7a**–**7h** to **9a**–**9h** were best performed in the presence of EDC × HCl as a coupling reagent (Scheme 1) to give products of high purity in good yields. Although we succeeded in purification of the phosphonate **7g** on a silica gel column, under the same conditions both homologs **8g** and **9g** were obtained as inseparable mixtures containing various amounts of triethylamine hydrochloride. When HPLC technique was applied, in both cases, partial deprotection of **8g** and **9g** occurred to provide pure mono-N-Boc protected phosphonates **8j** and **9j** (Fig. 3) in addition to mixtures of **8j** and **8g** as well as **9j** and **9g** free from triethylamine hydrochloride. Several attempts at transforming the 2-amino-6-chloropurine moiety in phosphonates **7h**–**9h** into guanine phosphonates **7i**–**9i** appeared fruitless leading to complex mixtures of unidentified products.

### Conformational analysis

**Fig. 4:**
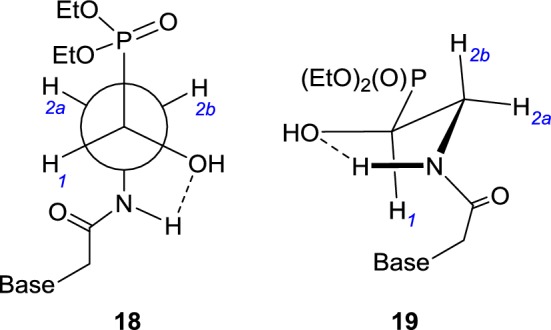
Preferred conformations **18**/**19** of nucleotide analogs **7a**–7**h**

We were also interested in the conformational mobility of the amides **7a**–**7h** to **9a**–**9h** within new acyclic linkers to provide important information regarding structure–activity analysis. Since ^1^H NMR spectra of all nucleotide analogs **7a**–**7h** exhibited almost identical patterns regarding a P–CH(OH)–CH_2_–NHC(O)–CH_2_ fragment, we concluded that in a methanolic solution they exist in the same conformation. Detailed analysis of a ^1^H NMR spectrum of **7d**, including a ^1^H{^31^P} one, clearly revealed an antiperiplanar disposition of the phosphoryl and amide groups projected as **18** (Fig. 4). Vicinal couplings H1–H2a (3.6 Hz) and H1–H2b (9.2 Hz) fit well into the ranges expected for the gauche and antiperiplanar arrangement of these protons [30]. Furthermore, values of both ^3^*J*(P–H2a/H2b) coupling constants are close (7.2 and 8.5 Hz). These values were well correlated with those characteristic of the gauche hydrogen–phosphorus relationship [31, 32]. The stability of the conformation **18** primarily results from the steric bulkiness of the substituents at C1 and C2 but may also be enforced by a C2–N–H·······O(H)–C1 hydrogen bond within a five-membered ring as depicted in **19** (Fig. 4). This suggestion finds support in a large downfield shift of the H2a proton (3.73 ppm) as compared with the H2b proton (3.38 ppm) which can only be observed when an amide C=O acts as a deshielding group. This is only possible when a C2–N–H·······O(H)–C1 hydrogen bond is strong enough to enable a free rotation around a C2–NH bond.

**Fig. 5:**
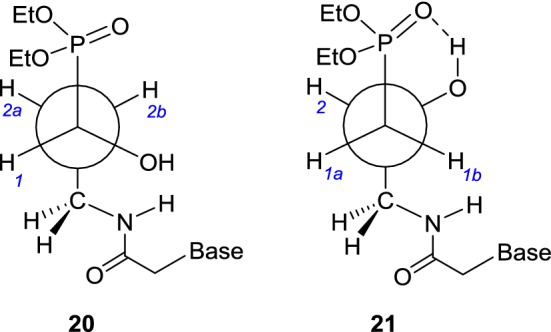
Preferred conformations **20** and **21** of nucleotide analogs **8a**–**8h** and **9a**–**9h**

Also, all ^1^H NMR spectra of nucleotide analogs **8a**–**8f** and **8h** showed almost identical patterns for a P–CH(OH)–CH_2_–CH_2_–NHC(O)–CH_2_ chain and we reasoned that in methanol they adopt the same conformation **20** (Fig. 5) which resembles **18** in terms of antiperiplanar location of the phosphoryl and H_2_C3 groups. From ^1^H, ^1^H{^31^P}, and ^13^C NMR spectra of **8c**, the following diagnostic coupling constants were extracted: ^3^*J*(H1–H2a) = 3.1 Hz, ^3^*J*(H1–H2b) = 10.7 Hz [30], ^3^*J*(P–H2a) = 6.7 Hz, ^3^*J*(P–H2b) = 9.5 Hz [31, 32], and ^3^*J*(P–C3) = 16.1 Hz [33, 34] which fully confirm our conclusion.

Thus, again the steric requirements of the diethoxyphosphoryl group and a H_2_C3–NHC(O) substituent are responsible for stability of the antiperiplanar conformation **20**. However, although all vicinal proton–proton couplings were successfully calculated for a H_a_H_b_C2–C3H_a_H_b_ subunit in **8c**, their values can only be interpreted in favor of a free rotation around a C2–C3 bond and thus preclude any intramolecular H-bonding for amide moieties in **8a**–**8h**.

Similar to ^1^H NMR spectra of nucleotide analogs **7a**–**7h** and **8a**–**8f** and **8h**, the spectra of the analogs **9a**–**9f** and **9h** in methanol displayed very similar patterns for a P–CH_2_–CH(OH)–CH_2_–NHC(O)–CH_2_ portion. The values of ^3^*J*(P–C3) = 15.4 Hz [33, 34] together with ^3^*J*(H1a–H2) = 4.6 Hz and ^3^*J*(H1b–H2) = 8.2 Hz [31, 32] found in ^13^C in ^1^H NMR spectra of **9a** again suggest that the diethoxyphosphoryl and CH_2_–NHC(O) groups for steric reasons prefer to exist in the *anti* conformation **21** (Fig. 5). This is commonly observed for *β*-hydroxyphosphonates [35]. This conclusion is further supported by the lack of the vicinal phosphorus–HC2 coupling as expected for the gauche relationship within a *H*-C2–C1-*P* unit [31, 32]. As in **8c**, a free rotation within a CH_2_–NHC(O) moiety in **9a** is evident from the values of vicinal H2–H3a/3b couplings.

### Antiviral evaluation

All phosphonates, i.e., **7a**–**7h**, **8a**–**8f**, **8h**, **8j**, **9a**–**9f**, **9h**, and **9j** were evaluated for inhibitory activity against a wide variety of DNA and RNA viruses, using the following cell-based assays. The antiviral assays were performed in: (a) human embryonic lung (HEL) cells [herpes simplex virus-1 (KOS strain), herpes simplex virus-2 (G strain), thymidine kinase deficient (acyclovir resistant) herpes simplex virus-1 (TK^–^ KOS ACV^r^ strain), vaccinia virus, adenovirus-2, human coronavirus (229E), cytomegalovirus (AD-169 and Davis strains), varicella-zoster virus (TK^+^ VZV and TK^–^ VZV strains)], (b) HeLa cell cultures (vesicular stomatitis virus, Coxsackie virus B4 and respiratory syncytial virus), (c) Vero cell cultures [para-influenza-3 virus, reovirus-1, Sindbis virus, Coxsackie virus B4, Punta Toro virus, yellow fever virus], and (d) MDCK cell cultures [influenza A virus (H1N1 and H3N2 subtypes) and influenza B virus]. Ganciclovir, cidofovir, acyclovir, brivudin, zalcitabine, zanamivir, alovudine, amantadine, rimantadine, ribavirin, dextran sulfate (molecular weight 10,000, DS-10000), mycophenolic acid and Urtica dioica agglutinin (UDA) were used as the reference compounds. The antiviral activity was expressed as the EC_50_: the compound concentration required to reduce virus plaque formation (VZV) by 50% or to reduce virus-induced cytopathogenicity by 50% (other viruses). None of the tested compounds showed appreciable antiviral activity toward any of the tested DNA and RNA viruses at concentrations up to 100 μM, nor affected cell morphology of HEL, HeLa, Vero, and MDCL cells.

### Cytostatic evaluation

The 50% cytostatic inhibitory concentration (IC_50_) causing a 50% decrease in cell proliferation was determined for all phosphonates, i.e., **7a**–**7h**, **8a**–**8f**, **8h**, **8j**, **9a**–**9f**, **9h**, and **9j** toward 9 cancerous cell lines [Capan-1 (pancreatic adenocarcinoma), Hap1 (chronic myeloid leukemia), HCT-116 (colorectal carcinoma), NCI-H460 (lung carcinoma), DND-41 (acute lymphoblastic leukemia), HL-60 (acute myeloid leukemia), K-562 (chronic myeloid leukemia), MM.1S (multiple myeloma), Z-138 non-Hodgkin lymphoma)] as well as normal retina (non cancerous) cells (hTERT RPE-1). Docetaxel and stauroporine were used as the reference compounds. Among all screened compounds, phosphonates **7c**, **8c**, and **9d** were slightly cytostatic against multiple myeloma cells (MM.1S) with IC_50_ values 78.8, 91.3, and 40.7 μM, respectively, however, lower than reference drugs (IC_50_ values 3.6 and 16.6 for docetaxel and stauroporine, respectively). On the other hand, compound **9b** showed noticeable inhibitory properties toward chronic myeloid leukemia (K-562) (IC_50_ = 28.8 μM), however, still lower than docetaxel (IC_50_ = 1.4 μM) and stauroporine (IC_50_ = 8.0 μM). All tested compounds were not toxic for normal, non-cancerous retina cells (hTERT RPE-1) at concentrations up to 100 μM.

## Conclusion

In further studies on a concept of the replacement of the P–CH_2_–**O**–**CHR**– fragment in acyclic nucleoside analogs (e.g., adefovir) for P–X–**HN**–**C**(**O**)– moieties in addition to previously obtained series (X=CH_2_, CH_2_CH_2_, CH_2_CH_2_CH_2_, CH_2_OCH_2_CH_2_), nucleotide analogs containing hydroxyalkyl linkers (X=CH(OH)CH_2_, CH(OH)CH_2_CH_2_, CH_2_CH(OH)CH_2_) were synthesized. The coupling of the respective ω-aminophosphonates and nucleobase-derived acetic acids was accomplished in good yields by application of EDC. Phosphonate–nucleobase linkers in the synthesized analogs contain two fragments of restricted rotation, namely the amide bonds and P–CH(OH)–CH_2_–N (compounds **7a**–**7h**), P–CH(OH)–CH_2_–C (compounds **8a**–**8h**), and P–CH_2_–CH(OH)–C (compounds **9a**–**9h**) residues in which, as judged from ^1^H and ^13^C NMR spectral data, antiperiplanar disposition of P and N/C atoms results both from the steric bulkiness of *O*,*O*-diethyl phosphoryl group and intramolecular H-bonding. The phosphonates **7a**–**7h**, **8a**–**8f**, **8h**, **8j**, **9a**–**9f**, **9h**, and **9j** were subjected to antiviral assays on a wide variety of DNA and RNA viruses but appeared inactive at concentrations up to 100 μM. Their cytostatic properties were evaluated on 9 cancerous cell lines and phosphonates **9b** and **9d** showed moderate activity towards myeloid leukemia (K-562) (IC_50_ = 28.8 μM) and multiple myeloma cells (MM.1S) (IC_50_ = 40.7 μM), respectively.

Studies on the analogous phosphonates containing functionalized amino groups within linkers are currently under way in this laboratory.

## Experimental

^1^H NMR spectra were recorded in CD_3_OD or CDCl_3_ on the following spectrometers: Varian Gemini 2000BB (200 MHz) and Bruker Avance III (600 MHz) with TMS as internal standard. ^13^C NMR spectra were recorder for CD_3_OD or CDCl_3_ solutions on the Bruker Avance III at 151.0 MHz. ^31^P NMR spectra were performed on the Varian Gemini 2000BB at 81.0 MHz or on Bruker Avance III at 243.0 MHz. IR spectral data were measured on a Bruker Alpha-T FT-IR spectrometer. Melting points were determined on a Boetius apparatus. Elemental analyses were performed by the Microanalytical Laboratory of this Faculty on a Perkin Elmer PE 2400 CHNS analyzer and their results were found to be in good agreement (± 0.3%) with the calculated values.

The following absorbents were used: column chromatography, Merck silica gel 60 (70–230 mesh); analytical TLC, Merck TLC plastic sheets silica gel 60 F_254_. TLC plates were developed in chloroform–methanol solvent systems. Visualization of spots was effected with iodine vapors. All solvents were purified by methods described in the literature.

Diethyl 1,2-epoxyethane- and 2,3-epoxypropanephosphonates **14** [36] and **15** [15] as well as the protected aldehyde **16** [20] and diethyl 3-amino-2-hydroxypropanephosphonate **12** [18] were prepared according to the literature procedures.

### Diethyl 2-amino-1-hydroxyethanephosphonate **10** [16]

A mixture of 3.60 g of the epoxide **14** (19.9 mmol) and 3.99 cm^3^ dibenzylamine (20.8 mmol) was heated at 60 °C for 20 h. After cooling, the crude product was purified on a silica gel column with a chloroform to give pure diethyl 2-(*N*,*N*-dibenzylamino)-1-hydroxyethanephosphonate (6.77 g) in 68% yield. In the next step a solution of 1.53 g of the diethyl 2-(*N*,*N*-dibenzylamino)-1-hydroxyethanephosphonate (4.05 mmol) in 10 cm^3^ ethanol was hydrogenated over 80 mg Pd–C (10%) at room temperature for 72 h. The catalyst was removed on a layer of Celite; the solution was concentrated in vacuo to afford pure phosphonate **10** (0.860 g, 100%) as a yellowish oil.

### Diethyl 3-amino-1-hydroxypropanephosphonate **11** [19]

A mixture of 2.02 g of the protected aldehyde **16** (9.76 mmol), 1.13 cm^3^ diethyl phosphite (8.78 mmol), and 0.136 cm^3^ triethylamine (0.976 mmol) was stirred at room temperature for 24 h. After the solution was concentrated in vacuo, the residue was chromatographed on a silica gel column with a chloroform–methanol mixture (200:1, 100:1 v/v) to give the phosphonate **17** (2.27 g, 67%) as a white powder. In the next step, a solution of 0.750 g of the phosphonate **17** (2.17 mmol) in 6 cm^3^ ethanol was hydrogenated over 30 mg Pd–C (10%) at room temperature for 72 h. The suspension was filtered through a pad of Celite and washed with ethanol. The solution was concentrated in vacuo to afford pure 3-amino-1-hydroxypropanephosphonate **11** (0.460 g, 100%) as a yellowish oil.

### General procedure

To a solution of aminophosphonates **10**–**12** (1.00 mmol) in 2 cm^3^ DMF or chloroform, the respective acetic acids **13a**–**13h** (1.00 mmol), EDC × HCl (1.00 mmol), and TEA (1.00 mmol) were added. The reaction mixture was stirred at room temperature for 48 h and then concentrated in vacuo. The residue was chromatographed on a silica gel column with chloroform–methanol mixtures and crystallized from the appropriate solvents.

#### Diethyl 2-[2-(3,4-dihydro-2,4-dioxopyrimidin-1(2*H*)-yl)acetamido]-1-hydroxyethylphosphonate (**7a**, C_12_H_20_N_3_O_7_P)

According to the general procedure from 0.156 g diethyl 2-amino-1-hydroxyethylphosphonate (**10**, 0.791 mmol) and 0.135 g (uracil-1-yl)acetic acid (**13a**, 0.791 mmol), the amide **7a** (0.128 g, 46% yield) was obtained as a white solid after purification on a silica gel column with chloroform–methanol mixtures (50:1, 20:1 v/v) and crystallization from methanol. M.p.: 174–176 °C; IR (KBr): $\overline{V}$ = 3340, 3229, 2997, 2940, 2824, 1687, 1046, 1015 cm^−1^; ^1^H NMR (200 MHz, CD_3_OD): *δ* = 7.55 (d, *J* = 7.9 Hz, 1H, *H*C = CH), 5.72 (d, *J* = 7.9 Hz, 1H, HC = C*H*), 4.50 and 4.48 (AB, *J* = 16.7 Hz, 2H, C(O)C*H*_a_*H*_b_), 4.31–4.13 (m, 4H, 2 × POC*H*_2_CH_3_), 4.08 (ddd, *J*_H1–H2b_ = 9.2 Hz, *J*_H1-P_ = 8.1 Hz, *J*_H1–H2a_ = 3.7 Hz, 1H, PC*H*CH_2_), 3.73 (ddd, *J*_H2a–H2b_ = 13.9 Hz, *J*_H2a-P_ = 7.2 Hz, *J*_H2a–H1_ = 3.7 Hz, 1H, PCC*H*_a_H_b_), 3.39 (ddd ~ dt, *J*_H2a–H2b_ = 13.9 Hz, *J*_H2b–H1_ = 9.2 Hz, *J*_H2b-P_ = 8.5 Hz, 1H, PCCH_a_*H*_b_), 1.39 and 1.38 (2 × t, *J* = 7.0 Hz, 2 × 3H, 2 × POCH_2_C*H*_3_) ppm; ^13^C NMR (151 MHz, CD_3_OD): *δ* = 168.26, 165.33, 151.55, 146.38, 100.97, 65.88 (d, *J* = 164.4 Hz, PC), 63.05 and 62.75 (2 × d, *J* = 7.3 Hz, 2 × POC), 49.72, 41.12 (d, *J* = 8.6 Hz, PC*C*), 15.40 and 15.38 (2 × d, *J* = 5.1 Hz, 2 × POC*C*) ppm; ^31^P NMR (81 MHz, CD_3_OD): *δ* = 23.73 ppm.

#### Diethyl 3-[2-(3,4-dihydro-2,4-dioxopyrimidin-1(2*H*)-yl)acetamido]-1-hydroxypropylphosphonate (**8a**, C_13_H_22_N_3_O_7_P)

According to the general procedure from 0.050 g diethyl 3-amino-1-hydroxypropylphosphonate (**11**, 0.240 mmol) and 0.040 g (uracil-1-yl)acetic acid (**13a**, 0.240 mmol), the amide **8a** (0.040 g, 47% yield) was obtained as a white solid after purification on a silica gel column with chloroform–methanol mixtures (50:1, 20:1 v/v) and crystallization from methanol–diethyl ether. M.p.: 190–191 °C; IR (KBr): $\overline{V}$ = 3333, 3231, 2995, 1698, 1673, 1244, 1028 cm^−1^; ^1^H NMR (200 MHz, CD_3_OD): *δ* = 7.56 (d, *J* = 8.0 Hz, 1H, *H*C = CH), 5.70 (d, *J* = 8.0 Hz, 1H, HC = C*H*), 4.45 (s, 2H, C(O)CH_2_), 4.29–4.12 (m, 4H, 2 × POC*H*_2_CH_3_), 3.98 (ddd, *J* = 10.6 Hz, *J* = 7.3 Hz, *J* = 3.4 Hz, 1H, PC*H*), 3.51–3.41 (m, 2H, PCCCH_2_), 2.11–1.73 (m, 2H, PCCH_2_), 1.38 (t, *J* = 7.1 Hz, 6H, 2 × POCH_2_C*H*_3_) ppm; ^13^C NMR (151 MHz, CD_3_OD): *δ* = 172.13, 169.35, 155.41, 151.31, 104.81, 68.25 (d, *J* = 167.3 Hz, PC), 66.83 and 66.49 (2 × d, *J* = 7.7 Hz, 2 × POC), 53.90, 39.61 (d, *J* = 16.4 Hz, PCC*C*), 34.73 (d, *J* = 3.4 Hz, PC*C*), 19.36 and 19.32 (2 × d, *J* = 5.2 Hz, 2 × POC*C*) ppm; ^31^P NMR (81 MHz, CD_3_OD): *δ* = 26.03 ppm.

#### Diethyl 3-[2-(3,4-dihydro-2,4-dioxopyrimidin-1(2*H*)-yl)acetamido]-2-hydroxypropylphosphonate (**9a**, C_13_H_22_N_3_O_7_P)

The crude product obtained from 0.139 g diethyl 3-amino-2-hydroxypropylphosphonate (**12**, 0.658 mmol) and 0.112 g (uracil-1-yl)acetic acid (**13a**, 0.658 mmol) according to the general procedure was chromatographed with chloroform–methanol mixtures (50:1, 20:1 v/v) and crystallized from a methanol–diethyl ether mixture to give compound **19a** (0.112 g, 47% yield) as a white powder. M.p.: 124–125 °C; IR (KBr): $\overline{V}$ = 3313, 3095, 3045, 2982, 2929, 1691, 1650, 1239, 1053, 1023 cm^−1^; ^1^H NMR (600 MHz, CD_3_OD): *δ* = 7.53 (d, *J* = 7.9 Hz, 1H, *H*C = CH), 5.69 (d, *J* = 7.9 Hz, 1H, HC = C*H*), 4.68 and 4.45 (AB, *J* = 16.6 Hz, 2H, C(O)C*H*_a_*H*_b_), 4.19–4.11 (m, 4H, 2 × POC*H*_2_CH_3_), 4.08 (ddt, *J* = 8.2 Hz, *J* = 6.6 Hz, *J* = 4.8 Hz, *J* = 4.6 Hz, 1H, PCC*H*), 3.42 (dd, *J* = 13.6 Hz, *J* = 4.8 Hz, 1H, PCCC*H*_a_H_b_), 3.29 (ddd, *J* = 13.6 Hz, *J* = 6.6 Hz, *J* = 0.6 Hz, 1H, PCCCH_a_*H*_b_), 2.07 (ddd, *J* = 18.9 Hz, *J* = 15.5 Hz, *J* = 4.6 Hz, 1H, PC*H*_a_H_b_), 2.01 (ddd, *J* = 17.9 Hz, *J* = 15.5 Hz, *J* = 8.2 Hz, 1H, PCH_a_*H*_b_), 1.36 and 1.35 (2 × t, *J* = 7.1 Hz, 2 × 3H, 2 × POCH_2_C*H*_3_) ppm; ^13^C NMR (151 MHz, CD_3_OD): *δ* = 168.32, 165.40, 151.51, 146.46, 100.88, 65.14 (d, *J* = 3.9 Hz, PC*C*), 62.06 and 61.84 (2 × d, *J* = 6.5 Hz, 2 × POC), 49.92, 45.83 (d, *J* = 15.4 Hz, PCC*C*), 30.56 (d, *J* = 140.7 Hz, PC), 15.26 (d, *J* = 6.2 Hz, 2 × POC*C*) ppm; ^31^P NMR (243 MHz, CD_3_OD): *δ* = 29.97 ppm.

#### Diethyl 2-[2-(5-fluoro-3,4-dihydro-2,4-dioxopyrimidin-1(2*H*)-yl)acetamido]-1-hydroxyethylphosphonate (**7b**, C_12_H_19_FN_3_O_7_P)

According to the general procedure from 0.107 g diethyl 2-amino-1-hydroxyethylphosphonate (**10**, 0.543 mmol) and 0.102 g (5-fluorouracil-1-yl)acetic acid (**13b**, 0.543 mmol), the amide **7b** (0.81 g, 41% yield) was obtained as a white powder after purification on a silica gel column with chloroform–methanol mixtures (50:1, 20:1 v/v). M.p.: 181–183 °C; IR (KBr): $\overline{V}$ = 3381, 3196, 3049, 2995, 2829, 1736, 1700, 1661, 1217, 1025, 749 cm^−1^; ^1^H NMR (200 MHz, CD_3_OD): *δ* = 7.82 (d, *J* = 6.2 Hz, 1H, *H*C = CF), 4.47 and 4.44 (AB, *J* = 16.6 Hz, 2H, C(O)C*H*_a_*H*_b_), 4.31–4.15 (m, 4H, 2 × POC*H*_2_CH_3_), 4.08 (ddd, *J* = 9.2 Hz, *J* = 8.2 Hz, *J* = 3.7 Hz, 1H, PC*H*CH_2_), 3.73 (ddd, *J* = 14.0 Hz, *J* = 6.9 Hz, *J* = 3.7 Hz, 1H, PCC*H*_a_H_b_), 3.45–3.33 (m, 1H, PCCH_a_*H*_b_), 1.39 (t, *J* = 7.0 Hz, 6H, 2 × POCH_2_C*H*_3_) ppm; ^13^C NMR (151 MHz, CD_3_OD): *δ* = 168.10, 158.51 (d, *J* = 25.6 Hz), 150.26, 140.23 (d, *J* = 232.3 Hz), 130.31 (d, *J* = 34.0 Hz), 65.84 (d, *J* = 164.8 Hz, PC), 63.05 and 62.76 (2 × d, *J* = 6.9 Hz, 2 × POC), 49.70, 41.11 (d, *J* = 8.6 Hz, PC*C*), 15.40 and 15.37 (2 × d, *J* = 5.0 Hz, 2 × POC*C*) ppm; ^31^P NMR (81 MHz, CD_3_OD): *δ* = 23.71 ppm.

#### Diethyl 3-[2-(5-fluoro-3,4-dihydro-2,4-dioxopyrimidin-1(2*H*)-yl)acetamido]-1-hydroxypropylphosphonate (**8b**, C_13_H_21_FN_3_O_7_P)

According to the general procedure from 0.160 g diethyl 3-amino-1-hydroxypropylphosphonate (**11**, 0.758 mmol) and 0.143 g (5-fluorouracil-1-yl)acetic acid (**13b**, 0.758 mmol), the amide **8b** (0.125 g, 43% yield) was obtained as a white powder after purification on a silica gel column with chloroform–methanol mixtures (50:1, 20:1 v/v) and crystallization from a methanol–diethyl ether mixture. M.p.: 211–212 °C; IR (KBr): $\overline{V}$ = 3336, 3217, 3061, 2929, 1721, 1698, 1659, 1245, 1019, 975 cm^−1^; ^1^H NMR (600 MHz, CD_3_OD): *δ* = 7.80 (d, *J* = 6.1 Hz, 1H, *H*C = CF), 4.42 and 4.38 (AB, *J* = 16.6 Hz, 2H, C(O)C*H*_a_*H*_b_), 4.23–4.16 (m, 4H, 2 × POC*H*_2_CH_3_), 3.96 (ddd, *J* = 10.7 Hz, *J* = 7.4 Hz, *J* = 3.1 Hz 1H, PC*H*CH_2_), 3.53–3.40 (m, 2H, PCCC*H*_2_), 2.03–1.96 (m, 1H, PCC*H*_a_CH_b_), 1.90–1.82 (m, 1H, PCCH_a_C*H*_b_), 1.36 (t, *J* = 7.1 Hz, 6H, 2 × POCH_2_C*H*_3_) ppm; ^13^C NMR (151 MHz, CD_3_OD): *δ* = 168.02, 158.57 (d, *J* = 25.6 Hz), 150.20, 140.22 (d, *J* = 232.0 Hz), 130.37 (d, *J* = 34.0 Hz), 64.37 (d, *J* = 166.1 Hz, P*C*), 62.90 and 62.55 (2 × d, *J* = 7.1 Hz, 2 × POC), 49.95, 35.69 (d, *J* = 16.1 Hz, PCC*C*), 30.78 (d, *J* = 3.0 Hz, PC*C*), 15.42 and 15.39 (2 × d, *J* = 5.3 Hz, 2 × POC*C*) ppm; ^31^P NMR (243 MHz, CD_3_OD): *δ* = 25.14 ppm.

#### Diethyl 3-[2-(5-fluoro-3,4-dihydro-2,4-dioxopyrimidin-1(2*H*)-yl)acetamido]-2-hydroxypropylphosphonate (**9b**, C_13_H_21_FN_3_O_7_P)

According to the general procedure from 0.128 g diethyl 3-amino-2-hydroxypropylphosphonate (**12**, 0.606 mmol) and 0.144 g (5-fluorouracil-1-yl)acetic acid (**13b**, 0.606 mmol), the amide **9b** (0.074 g, 32% yield) was obtained as a white powder after purification on a silica gel column with chloroform–methanol mixtures (50:1, 20:1 v/v) and HPLC (C18 column, 25:70 methanol:water). M.p.: 126–128 °C; IR (KBr): $\bar{V}$ = 3358, 3167, 3057, 2995, 2931, 2819, 1706, 1661, 1220, 1027, 858 cm^−1^; ^1^H NMR (600 MHz, CD_3_OD): *δ* = 7.80 (d, *J* = 6.2 Hz, 1H, *H*C = CF), 4.44 and 4.42 (AB, *J* = 16.8 Hz, 2H, C(O)C*H*_a_*H*_b_), 4.18–4.11 (m, 4H, 2 × POC*H*_2_CH_3_), 4.10–4.05 (m, 1H, PCC*H*), 3.43 (dd, *J* = 13.6 Hz, *J* = 4.7 Hz, 1H, PCCC*H*_a_H_b_), 3.28 (ddd, *J* = 13.6 Hz, *J* = 6.5 Hz, *J* = 0.5 Hz, 1H, PCCCH_a_*H*_b_), 2.08 (ddd, *J* = 19.0 Hz, *J* = 15.5 Hz, *J* = 4.7 Hz, 1H, PC*H*_a_H_b_), 2.01 (ddd, *J* = 17.8 Hz, *J* = 15.5 Hz, *J* = 8.2 Hz, 1H, PCH_a_*H*_b_), 1.36 and 1.35 (2 × t, *J* = 7.1 Hz, 2 × 3H, 2 × POCH_2_C*H*_3_) ppm; ^13^C NMR (151 MHz, CD_3_OD): *δ* = 168.17, 158.67 (d, *J* = 26.0 Hz), 150.33, 140.23 (d, *J* = 232.2 Hz), 130.35 (d, *J* = 34.0 Hz), 65.13 (d, *J* = 3.5 Hz, PC*C*), 62.07 and 61.84 (2 × d, *J* = 6.5 Hz, 2 × POC), 49.91, 45.83 (d, *J* = 15.3 Hz, PCC*C*), 30.59 (d, *J* = 140.9 Hz, PC), 15.26 (d, *J* = 6.0 Hz, 2 × POC*C*) ppm; ^31^P NMR (243 MHz, CD_3_OD): *δ* = 29.95 ppm.

#### Diethyl 2-[2-(5-bromo-3,4-dihydro-2,4-dioxopyrimidin-1(2*H*)-yl)acetamido]-1-hydroxyethylphosphonate (**7c**, C_12_H_19_BrN_3_O_7_P)

According to the general procedure from 0.177 g diethyl 2-amino-1-hydroxyethylphosphonate (**10**, 0.898 mmol) and 0.224 g (5-bromouracil-1-yl)acetic acid (**13c**, 0.898 mmol), the amide **7c** (0.169 g, 44% yield) was obtained as a white solid after purification on a silica gel column with chloroform–methanol mixtures (50:1, 20:1 v/v) and crystallization from a methanol–diethyl ether mixture. M.p.: 183–185 °C; IR (KBr): $\overline{V}$ = 3317, 3234, 3042, 2997, 2833, 1692, 1626, 1225, 1021, 622 cm^−1^; ^1^H NMR (600 MHz, CD_3_OD): *δ* = 8.00 (s, 1H, *H*C = CBr), 4.50 and 4.47 (AB, *J* = 16.4 Hz, 2H, C(O)C*H*_a_*H*_b_), 4.24–4.18 (m, 4H, 2 × POC*H*_2_CH_3_), 4.07 (dt, *J* = 8.8 Hz, *J* = 3.7 Hz, 1H, PC*H*CH_2_), 3.71 (ddd, *J* = 13.9 Hz, *J* = 7.4 Hz, *J* = 3.7 Hz, 1H, PCC*H*_a_H_b_), 3.38 (dt, *J* = 13.9 Hz, *J* = 8.8 Hz, 1H, PCCH_a_*H*_b_), 1.37 (t, *J* = 7.0 Hz, 6H, 2 × POCH_2_C*H*_3_) ppm; ^13^C NMR (151 MHz, CD_3_OD): *δ* = 168.03, 160.71, 150.96, 145.81, 95.18, 65.83 (d, *J* = 164.8 Hz, PC), 63.04 and 62.76 (2 × d, *J* = 7.1 Hz, 2 × POC), 49.73, 41.12 (d, *J* = 8.6 Hz, PC*C*), 15.40 and 15.38 (2 × d, *J* = 5.1 Hz, 2 × POC*C*) ppm; ^31^P NMR (81 MHz, CD_3_OD): *δ* = 23.71 ppm.

#### Diethyl 3-[2-(5-bromo-3,4-dihydro-2,4-dioxopyrimidin-1(2*H*)-yl)acetamido]-1-hydroxypropylphosphonate (**8c**, C_13_H_21_BrN_3_O_7_P)

According to the general procedure from 0.160 g diethyl 3-amino-1-hydroxypropylphosphonate (**11**, 0.758 mmol) and 0.160 g (5-bromouracil-1-yl)acetic acid (**13c**, 0.758 mmol), the amide **8c** (0.127 g, 44% yield) was obtained as a white powder after purification on a silica gel column with chloroform–methanol mixtures (50:1, 20:1 v/v). M.p.: 203–205 °C; IR (KBr): $\bar{V}$ = 3326, 3189, 3059, 2997, 1725, 1674, 1247, 1021, 586 cm^−1^; ^1^H NMR (600 MHz, CD_3_OD): *δ* = 8.01 (s, 1H, *H*C = CBr), 4.45 (s, 2H, C(O)CH_2_), 4.23–4.16 (m, 4H, 2 × POC*H*_2_CH_3_,), 3.96 (ddd, *J*_H1–H2b_ = 10.7 Hz, *J*_H1-P_ = 7.4 Hz, *J*_H1–H2a_ = 3.1 Hz, 1H, PC*H*) 3.48 (ddd, *J*_H3a–H3b_ = 13.5 Hz, *J*_H3a–H2a_ = 8.2 Hz, *J*_H2a-P_ = 7.0 Hz, 1H, PCCC*H*_a_H_b_), 3.41 (ddd, *J*_H3a–H3b_ = 13.5 Hz, *J*_H3b–H2a_ = 7.3 Hz, *J*_H3b–H2b_ = 4.9 Hz, 1H, PCCCH_a_*H*_b_), 1.99 (ddddd, *J*_H2a–H2b_ = 14.0 Hz, *J*_H2a–H3a_ = 8.2 Hz, *J*_H2a–H3b_ = 7.3 Hz, *J*_H2a-P_ = 6.7 Hz, *J*_H2a–H1_ = 3.1 Hz, 1H, PCC*H*_a_H_b_), 1.86 (ddddd, *J*_H2a–H2b_ = 14.0 Hz, *J*_H2b–H1_ = 10.7 Hz, *J*_H2b-P_ = 9.5 Hz, *J*_H2b–H3a_ = 7.0 Hz, *J*_H2b–H3b_ = 4.9 Hz, 1H, PCCH_a_*H*_b_) 1.36 (t, *J* = 7.1 Hz, 6H, 2 × POCH_2_C*H*_3_) ppm; ^13^C NMR (151 MHz, CD_3_OD): *δ* = 167.95, 160.77, 150.89, 145.86, 95.13, 64.35 (d, *J* = 167.4 Hz, PC), 62.90 and 62.56 (2 × d, *J* = 7.1 Hz, 2 × POC), 49.99, 35.68 (d, *J* = 16.1 Hz, PCC*C*), 30.78 (d, *J* = 3.0 Hz, PC*C*), 15.42 and 15.38 (2 × d, *J* = 4.8 Hz, 2 × POC*C*) ppm; ^31^P NMR (243 MHz, CD_3_OD): *δ* = 25.15 ppm.

#### Diethyl 3-[2-(5-bromo-3,4-dihydro-2,4-dioxopyrimidin-1(2*H*)-yl)acetamido]-2-hydroxypropylphosphonate (**9c**, C_13_H_21_BrN_3_O_7_P)

According to the general procedure from 0.545 g diethyl 3-amino-2-hydroxypropylphosphonate (**12**, 2.58 mmol) and 0.642 g (5-bromouracil-1-yl)acetic acid (**13c**, 2.58 mmol), the amide **9c** (0.552 g, 48% yield) was obtained as a white powder after purification on a silica gel column with chloroform–methanol mixtures (50:1, 20:1 v/v) and HPLC (C18 column, 25:70 methanol:water). M.p.: 108–109 °C; IR (KBr): $\overline{V}$ = 3488, 3350, 3159, 3034, 2993, 2928, 2838, 1714, 1668, 1240, 1021, 632 cm^−1^; ^1^H NMR (600 MHz, CDCl_3_): *δ* = 7.73 (brs, 2H, *H*C = CBr, NH), 4.47 (s, 2H, C(O)CH_2_), 4.22–4.11 (m, 5H, 2 × POC*H*_2_CH_3_, PCC*H*), 3.56–3.53 (m, 1H, PCCC*H*_a_H_b_), 3.30 (dt, *J* = 13.1 Hz, *J* = 6.4 Hz, 1H, PCCCH_a_*H*_b_), 2.09–1.97 (m, 2H, PC*H*_2_), 1.35 and 1.34 (2 × t, *J* = 7.0 Hz, 2 × 3H, 2 × POCH_2_C*H*_3_) ppm; ^13^C NMR (151 MHz, CDCl_3_): *δ* = 167.20, 160.14, 151.06, 144.87, 96.72, 65.73 (d, *J* = 3.1 Hz, PC*C*), 62.33 and 62.27 (2 × d, *J* = 6.6 Hz, 2 × POC), 50.67, 46.13 (d, *J* = 17.3 Hz, PCC*C*), 30.93 (d, *J* = 140.4 Hz, PC), 16.39 and 16.36 (2 × d, *J* = 6.1 Hz, 2 × POC*C*) ppm; ^31^P NMR (243 MHz, CDCl_3_): *δ* = 29.23 ppm.

#### Diethyl 2-[2-(3,4-dihydro-5-methyl-2,4-dioxopyrimidin-1(2*H*)-yl)acetamido]-1-hydroxyethylphosphonate (**7d**, C_13_H_22_N_3_O_7_P)

According to the general procedure from 0.158 g diethyl 2-amino-1-hydroxyethylphosphonate (**10**, 0.801 mmol) and 0.148 g (thymine-1-yl)acetic acid (**13d**, 0.801 mmol), the amide **7d** (0.145 g, 50% yield) was obtained as a white solid after purification on a silica gel column with chloroform–methanol mixtures (50:1, 20:1 v/v) and crystallization from a methanol–ethyl acetate mixture. M.p.: 186–187 °C; IR (KBr): $\overline{V}$ = 3318, 3261, 3043, 2943, 2910, 2830, 1697, 1653, 1245, 1018 cm^−1^; ^1^H NMR (200 MHz, CD_3_OD): *δ* = 7.39 (q, *J* = 1.1 Hz, 1H, *H*C = CCH_3_), 4.47 and 4.44 (AB, *J* = 16.6 Hz, 2H, C(O)C*H*_a_*H*_b_), 4.31–4.14 (m, 4H, 2 × POC*H*_2_CH_3_), 4.08 (ddd, *J*_H1–H2b_ = 9.2 Hz, *J*_H1-P_ = 8.2 Hz, *J*_H1–H2a_ = 3.6 Hz, 1H, PC*H*CH_2_), 3.73 (ddd, *J*_H2a–H2b_ = 14.0 Hz, *J*_H2a-P_ = 7.2 Hz, *J*_H2a–H1_ = 3.6 Hz, 1H, PCC*H*_a_H_b_), 3.38 (ddd ~ dt, *J*_H2b–H2a_ = 14.0 Hz, *J*_H2b–H1_ = 9.2 Hz, *J*_H2b-P_ = 8.5 Hz, 1H, PCCH_a_*H*_b_), 1.92 (d, *J* = 1.1 Hz, 3H, HC = CC*H*_3_), 1.39 (t, *J* = 7.0 Hz, 6H, 2 × POCH_2_C*H*_3_) ppm; ^13^C NMR (151 MHz, CD_3_OD): *δ* = 168.45, 165.54, 151.72, 142.17, 109.78, 65.84 (d, *J* = 164.3 Hz, PC), 63.04 and 62.73 (2 × d, *J* = 6.9 Hz, 2 × POC), 49.56, 41.11 (d, *J* = 8.6 Hz, PC*C*), 15.40 and 15.36 (2 × d, *J* = 5.1 Hz, 2 × POC*C*), 10.78 ppm; ^31^P NMR (81 MHz, CD_3_OD): *δ* = 23.74 ppm.

#### Diethyl 3-[2-(3,4-dihydro-5-methyl-2,4-dioxopyrimidin-1(2*H*)-yl)acetamido]-1-hydroxypropylphosphonate (**8d**, C_14_H_24_N_3_O_7_P)

According to the general procedure from 0.180 g diethyl 3-amino-1-hydroxypropylphosphonate (**11**, 0.852 mmol) and 0.157 g (thymine-1-yl)acetic acid (**13d**, 0.852 mmol), the amide **8d** (0.166 g, 52% yield) was obtained as a white solid after purification on a silica gel column with chloroform–methanol mixtures (50:1, 20:1 v/v) and crystallization from a methanol–diethyl ether mixture. M.p.: 183–184 °C; IR (KBr): $\bar{V}$ = 3333, 3238, 2987, 2833, 1665, 1236, 1024 cm^−1^; ^1^H NMR (600 MHz, CD_3_OD): *δ* = 7.38 (brq, *J* = 0.8 Hz, 1H, *H*C = CCH_3_), 4.41 and 4.39 (AB, *J* = 16.6 Hz, 2H, C(O)C*H*_a_*H*_b_), 4.22–4.16 (m, 4H, 2 × POC*H*_2_CH_3_), 3.96 (ddd, *J* = 10.6 Hz, *J* = 7.4 Hz, *J* = 3.1 Hz, 1H, PC*H*CH_2_), 3.49–3.39 (m, 2H, PCCC*H*_a_*H*_b_), 2.02–1.95 (m, 1H, PCC*H*_a_H_b_), 1.90–1.81 (m, 1H, PCCH_a_*H*_b_), 1.90 (d, *J* = 0.8 Hz, 3H, HC = CC*H*_3_), 1.36 (t, *J* = 7.1 Hz, 6H, 2 × POCH_2_C*H*_3_) ppm; ^13^C NMR (151 MHz, CD_3_OD): *δ* = 168.37, 165.60, 151.64, 142.23, 109.68, 64.38 (d, *J* = 167.3 Hz, PC), 62.88 and 62.54 (2 × d, *J* = 7.2 Hz, 2 × POC), 49.79, 35.66 (d, *J* = 15.9 Hz, PCC*C*), 30.78 (d, *J* = 2.7 Hz, PC*C*),15.41 and 15.36 (2 × d, *J* = 4.8 Hz, 2 × POC*C*), 10.78 ppm; ^31^P NMR (243 MHz, CD_3_OD): *δ* = 25.14 ppm.

#### Diethyl 3-[2-(3,4-dihydro-5-methyl-2,4-dioxopyrimidin-1(2*H*)-yl)acetamido]-2-hydroxypropylphosphonate (**9d**, C_14_H_24_N_3_O_7_P)

According to the general procedure from 0.127 g diethyl 3-amino-2-hydroxypropylphosphonate (**12**, 0.601 mmol) and 0.111 g (thymine-1-yl)acetic acid (**13d**, 0.601 mmol), the amide **9d** (0.113 g, 47% yield) was obtained as a white solid after purification on a silica gel column with chloroform–methanol mixtures (50:1, 20:1 v/v) and crystallization from a methanol–ethyl acetate mixture. M.p.: 157–158 °C; IR (KBr): $\overline{V}$ = 3428, 3186, 3057, 2991, 2936, 2828, 1697, 1651, 1225, 1030 cm^−1^; ^1^H NMR (600 MHz, CD_3_OD): *δ* = 7.38 (q, *J* = 1.0 Hz, 1H, *H*C = CCH_3_), 4.43 (s, 2H, C(O)CH_2_), 4.17–4.13 (m, 4H, 2 × POC*H*_2_CH_3_), 4.12–4.08 (m, 1H, PCC*H*), 3.42 (dd, *J* = 13.6 Hz, *J* = 4.7 Hz, 1H, PCCC*H*_a_H_b_), 3.28 (dd, *J* = 13.6 Hz, *J* = 6.7 Hz, 1H, PCCCH_a_*H*_b_), 2.08 (ddd, *J* = 18.9 Hz, *J* = 15.5 Hz, *J* = 4.7 Hz, 1H, PC*H*_a_H_b_), 2.01 (ddd, *J* = 17.8 Hz, *J* = 15.5 Hz, *J* = 7.4 Hz, 1H, PCH_a_*H*_b_), 1.90 (d, *J* = 1.0 Hz, 3H, HC = CC*H*_3_), 1.36 and 1.35 (2 × t, *J* = 7.1 Hz, 2 × 3H, 2 × POCH_2_C*H*_3_) ppm;^13^C NMR (151 MHz, CD_3_OD): *δ* = 168.51, 165.57, 151.68, 142.26, 109.67, 65.13 (d, *J* = 3.6 Hz, PC*C*), 62.06 and 61.83 (2 × d, *J* = 6.4 Hz, 2 × POC), 49.76, 45.83 (d, *J* = 15.4 Hz, PCC*C*), 30.57 (d, *J* = 140.7 Hz, PC), 15.26 (d, *J* = 6.3 Hz, 2 × POC*C*) ppm; ^31^P NMR (243 MHz, CD_3_OD): *δ* = 29.97 ppm.

#### Diethyl 2-[2-[4-[(benzyloxycarbonyl)amino]-2-oxopyrimidin-1(2*H*)-yl]acetamido]-1-hydroxyethylphosphonate (**7e**, C_20_H_27_N_4_O_8_P)

According to the general procedure from 0.090 g diethyl 2-amino-1-hydroxyethylphosphonate (**10**, 0.456 mmol) and 0.138 g [*N*^4^-(benzyloxycarbonyl)cytosine-1-yl]acetic acid (**13e**, 0.456 mmol), the amide **7e** (0.074 g, 34% yield) was obtained as a colorless oil after purification on a silica gel column with chloroform–methanol mixtures (50:1, 20:1 v/v). IR (film): $\overline{V}$ = 3279, 3088, 2984, 2931, 1749, 1661, 1215, 1023 cm^−1^; ^1^H NMR (200 MHz, CDCl_3_): *δ* = 10.39 (brs, 1H, NH), 8.75 (brt, 1H, NH), 7.72 (d, *J* = 7.4 Hz, 1H, *H*C = CCH), 7.38–7.28 (m, 6H, C_6_H_5_, *H*C = CCH), 5.85 (brs, 1H, NH), 5.21 (s, 2H, C*H*_2_C_6_H_5_), 4.59 and 4.48 (AB, *J* = 14.9 Hz, 2H, C(O)C*H*_a_*H*_b_), 4.20–3.82 (m, 6H, 2 × POC*H*_2_CH_3_, PC*H*C*H*_*a*_H_b_), 3.58–3.38 (m, 1H, PCCH_a_*H*_*b*_), 1.31 and 1.20 (2 × t, *J* = 7.0 Hz, 2 × 3H, 2 × POCH_2_C*H*_3_) ppm;^13^C NMR (151 MHz, CDCl_3_): *δ* = 166.81, 163.67, 156.06, 153.04, 149.30, 135.44, 128.53, 128.37, 128.01, 95.99, 67.54, 66.89 (d, *J* = 161.6 Hz, P*C*), 63.47 and 62.55 (2 × d, *J* = 6.8 Hz, 2 × POC), 52.45, 40.79 (d, *J* = 10.1 Hz, PC*C*), 16.41 (2 × d, *J* = 5.4 Hz, 2 × POC*C*) ppm; ^31^P NMR (81 MHz, CDCl_3_): *δ* = 24.42 ppm.

#### Diethyl 3-[2-[4-[(benzyloxycarbonyl)amino]-2-oxopyrimidin-1(2*H*)-yl]acetamido]-1-hydroxypropylphosphonate (**8e**, C_21_H_29_N_4_O_8_P × 0.5H_2_O)

According to the general procedure from 0.166 g diethyl 3-amino-1-hydroxypropylphosphonate (**11**, 0.787 mmol) and 0.239 g [*N*^4^-(benzyloxycarbonyl)cytosine-1-yl]acetic acid (**13e**, 0.787 mmol), the amide **8e** (0.248 g, 64% yield) was obtained as a white solid after crystallization from a methanol–diethyl ether mixture. M.p.: 164–165 °C; IR (KBr): $\bar{V}$ = 3291, 3093, 2977, 1753, 1689, 1657, 1226, 1027 cm^−1^; ^1^H NMR (600 MHz, CD_3_OD): *δ* = 7.93 (d, *J* = 7.3 Hz, 1H, *H*C = CCH), 7.45–7.31 (m, 6H, C_6_H_5_, *H*C = CCH), 5.25 (s, 2H, C*H*_2_C_6_H_5_), 4.59 and 4.54 (AB, *J* = 15.7 Hz, 2H, C(O)C*H*_a_*H*_b_), 4.22–4.15 (m, 4H, 2 × POC*H*_2_CH_3_), 3.97 (ddd, *J* = 10.7 Hz, *J* = 7.4 Hz, *J* = 3.1 Hz, 1H, PC*H*CH_2_), 3.50–3.40 (m, 2H, PCCC*H*_2_), 2.09–1.96 (m, 1H, PCC*H*_a_H_b_), 1.92–1.82 (m, 1H, PCCH_a_*H*_b_), 1.36 (t, *J* = 7.1 Hz, 6H, 2 × POCH_2_C*H*_3_) ppm; ^13^C NMR (151 MHz, CD_3_OD): *δ* = 167.91, 164.05, 150.18, 135.81, 128.20, 128.20, 128.04, 127.89, 127.89, 95.35, 67.20, 64.35 (d, *J* = 167.4 Hz, P*C*), 62.88 and 61.54 (2 × d, *J* = 7.3 Hz, 2 × POC), 52.01, 35.71 (d, *J* = 16.2 Hz, PCC*C*), 30.75 (d, *J* = 2.7 Hz, PC*C*), 15.42 and 15.38 (2 × d, *J* = 4.8 Hz, 2 × POC*C*) ppm; ^31^P NMR (243 MHz, CD_3_OD): *δ* = 25.15 ppm.

#### Diethyl 3-[2-[4-[(benzyloxycarbonyl)amino]-2-oxopyrimidin-1(2*H*)-yl]acetamido]-2-hydroxypropylphosphonate (**9e**, C_21_H_29_N_4_O_8_P × 0.5H_2_O)

According to the general procedure from 0.119 g diethyl 3-amino-2-hydroxypropylphosphonate (**12**, 0.563 mmol) and 0.171 g [*N*^4^-(benzyloxycarbonyl)cytosine-1-yl]acetic acid (**13e**, 0.563 mmol), the amide **9e** (0.100 g, 36% yield) was obtained as a white solid after purification on a silica gel column with chloroform–methanol mixtures (50:1, 20:1 v/v) and crystallization from ethanol. M.p.: 123–125 °C; IR (KBr): $\overline{V}$ = 3268, 3088, 2981, 2931, 1746, 1666, 1661, 1218, 1027 cm^−1^; ^1^H NMR (200 MHz, CD_3_OD): *δ* = 7.96 (d, *J* = 7.4 Hz, 1H, *H*C = CCH), 7.52–7.33 (m, 6H, C_6_H_5_, *H*C = CCH), 5.27 (s, 2H, C*H*_2_C_6_H_5_), 4.62 (s, 2H, C(O)CH_2_), 4.25–4.02 (m, 5H, 2 × POC*H*_2_CH_3_, PCC*H*), 3.46 (dd, *J* = 13.9 Hz, *J* = 4.7 Hz, 1H, PCCC*H*_a_H_b_), 3.25 (dd, *J* = 13.9 Hz, *J* = 7.3 Hz, 1H, PCCCH_a_*H*_b_), 2.21–1.91 (m, 2H, PCH_2_), 1.36 (t, *J* = 7.1 Hz, 6H, 2 × POCH_2_C*H*_3_) ppm; ^13^C NMR (151 MHz, CD_3_OD): *δ* = 168.05, 164.03, 157.12, 153.16, 150.26, 135.80, 128.21, 128.21, 128.05, 127.89, 127.89, 90.37, 67.21, 65.14 (d, *J* = 3.6 Hz, PC*C*), 62.05 and 61.83 (2 × d, *J* = 6.4 Hz, 2 × POC), 52.02, 45.93 (d, *J* = 15.4 Hz, PCC*C*), 30.58 (d, *J* = 140.7 Hz, PC), 15.29 (d, *J* = 6.0 Hz, 2 × POC*C*) ppm; ^31^P NMR (81 MHz, CD_3_OD): *δ* = 30.88 ppm.

#### Diethyl 2-[2-(3,4-dihydro-2,4-dioxoquinazolin-1(2*H*)-yl)acetamido]-1-hydroxyethylphosphonate (**7f**, C_16_H_22_N_3_O_7_P)

According to the general procedure from 0.158 g diethyl 2-amino-1-hydroxyethylphosphonate (**10**, 0.801 mmol) and 0.176 g (3,4-dihydro-2,4-dioxoquinazolin-1-yl)acetic acid (**13f**, 0.801 mmol), the pure amide **7f** (0.211 g, 66% yield) was obtained as a white powder after a mere filtration of the reaction mixture. M.p.: 225–226 °C; IR (KBr): $\overline{V}$ = 3248, 3070, 2986, 2935, 1735, 1637, 1040 cm^−1^; ^1^H NMR (600 MHz, CD_3_OD): *δ* = 8.06 (dd, *J* = 7.9 Hz, *J* = 1.0 Hz, 1H, H_aromat_.), 7.69 (dt, *J* = 8.4 Hz, *J* = 1.0 Hz, 1H, H_aromat_.), 7.27 (dt, *J* = 7.9 Hz, *J* = 0.5 Hz, 1H, H_aromat_.), 7.22 (d, *J* = 8.4 Hz, 1H, H_aromat_.), 4.76 and 4.70 (AB, *J* = 16.0 Hz, 2H, C(O)C*H*_a_*H*_b_), 4.24–4.18 (m, 4H, 2 × POC*H*_2_CH_3_), 4.08 (dt, *J* = 9.2 Hz, *J* = 3.7 Hz, 1H, PC*H*CH_2_), 3.73 (ddd, *J* = 13.9 Hz, *J* = 6.2 Hz, *J* = 3.7 Hz, 1H, PCC*H*_a_H_b_), 3.37–3.31 (m, 1H, PCCH_a_*H*_b_), 1.37 (t, *J* = 7.0 Hz, 6H, 2 × POCH_2_C*H*_3_) ppm; ^13^C NMR (151 MHz, CD_3_OD): *δ* = 166.87, 162.78, 150.70, 139.59, 135.06, 127.52, 122.75, 114.90, 114.02, 65.90 (d, *J* = 164.6 Hz, PC), 63.00 and 62.73 (2 × d, *J* = 6.6 Hz, 2 × POC), 42.44, 41.16 (d, *J* = 8.8 Hz, PC*C*), 15.38 and 15.37 (2 × d, *J* = 5.2 Hz, 2 × POC*C*) ppm; ^31^P NMR (81 MHz, CD_3_OD): *δ* = 23.05 ppm.

#### Diethyl 3-[2-(3,4-dihydro-2,4-dioxoquinazolin-1(2*H*)-yl)acetamido]-1-hydroxypropylphosphonate (**8f**, C_17_H_24_N_3_O_7_P)

According to the general procedure from 0.180 g diethyl 3-amino-1-hydroxypropylphosphonate (**11**, 0.852 mmol) and 0.187 g (3,4-dihydro-2,4-dioxoquinazolin-1-yl)acetic acid (**13f**, 0.852 mmol), the amide **8f** (0.168 g, 48% yield) was obtained as a white powder after purification on a silica gel column with chloroform–methanol mixtures (50:1, 20:1 v/v) and crystallization from an ethanol–diethyl ether mixture. M.p.: 205–208 °C; IR (KBr): $\overline{V}$ = 3256, 3205, 3075, 2984, 2954, 1736, 1654, 1635, 1024 cm^−1^; ^1^H NMR (600 MHz, CD_3_OD): *δ* = 8.01(d, *J* = 7.8 Hz, 1H, H_aromat_.), 7.69 (dt, *J* = 8.4 Hz, *J* = 1.2 Hz, 1H, H_aromat_.), 7.27 (t, *J* = 7.8 Hz, 1H, H_aromat_.), 7.22 (d, *J* = 8.3 Hz, 1H, H_aromat_.), 4.71 and 4.68 (AB, *J* = 16.0 Hz, 2H, C(O)C*H*_a_*H*_b_), 4.23–4.16 (m, 4H, 2 × POC*H*_2_CH_3_), 3.99 (ddd, *J* = 10.7 Hz, *J* = 7.7 Hz, *J* = 3.0 Hz, 1H, PC*H*CH_2_), 3.52–3.47 (m, 1H, PCCC*H*_a_H_b_), 3.41–3.37 (m, 1H, PCCCH_a_*H*_b_), 2.03–1.96 (m, 1H, PCC*H*_a_H_b_), 1.89–1.81 (m, 1H, PCCH_a_*H*_b_), 1.35 (t, *J* = 7.0 Hz, 6H, 2 × POCH_2_C*H*_3_) ppm; ^13^C NMR (151 MHz, CD_3_OD): *δ* = 168.91, 162.84, 150.86, 139.61, 135.03, 127.51, 122.51, 114.88, 114.06, 64.21 (d, *J* = 167.3 Hz, PC), 62.86 and 62.53 (2 × d, *J* = 7.1 Hz, 2 × POC), 42.56, 35.49 (d, *J* = 16.0 Hz, PCC*C*), 30.84 (d, *J* = 3.0 Hz, PC*C*), 15.41 and 15.38 (2 × d, *J* = 5.2 Hz, 2 × POC*C*) ppm; ^31^P NMR (243 MHz, CD_3_OD): *δ* = 25.29 ppm.

#### Diethyl 3-[2-(3,4-dihydro-2,4-dioxoquinazolin-1(2*H*)-yl)acetamido]-2-hydroxypropylphosphonate (**9f**, C_17_H_24_N_3_O_7_P)

According to the general procedure from 0.127 g diethyl 3-amino-2-hydroxypropylphosphonate (**12**, 0.601 mmol) and 0.132 g (3,4-dihydro-2,4-dioxoquinazolin-1-yl)acetic acid (**13f**, 0.601 mmol), the amide **9f** (0.131 g, 53% yield) was obtained as a white solid after crystallization from an ethanol–diethyl ether mixture. M.p.: 168–169 °C; IR (KBr): $\bar{V}$ = 3378, 3249, 3201, 2985, 2946, 1734, 1656, 1638, 1020 cm^−1^; ^1^H NMR (200 MHz, CD_3_OD): *δ* = 8.08 (ddd, *J* = 8.0 Hz, *J* = 1.0 Hz, *J* = 0.5 Hz, 1H, H_aromat_.), 7.71 (ddd, *J* = 8.2 Hz, *J* = 7.3 Hz, *J* = 1.5 Hz, 1H, H_aromat_.), 7.32–7.20 (m, 2H, H_aromat_.), 4.74 (s, 2H, C(O)CH_2_), 4.24–4.03 (m, 5H, 2 × POC*H*_2_CH_3_, PCC*H*), 3.44 (dd, *J* = 13.5 Hz, *J* = 5.1 Hz, 1H, PCCC*H*_a_H_b_), 3.31 (ddd, *J* = 13.5 Hz, *J* = 6.5 Hz, *J* = 1.6 Hz, 1H, PCCCH_a_*H*_b_), 2.13 (ddd, *J* = 18.8 Hz, *J* = 15.5 Hz, *J* = 4.6 Hz, 1H, PC*H*_a_H_b_), 1.99 (ddd, *J* = 17.7 Hz, *J* = 15.5 Hz, *J* = 8.2 Hz, 1H, PCH_a_*H*_b_), 1.36 (t, *J* = 7.1 Hz, 6H, 2 × POCH_2_C*H*_3_) ppm; ^13^C NMR (151 MHz, CD_3_OD): *δ* = 168.98, 162.83, 150.87, 139.60, 135.04, 127.51, 122.71, 114.89, 114.05, 65.24 (d, *J* = 4.2 Hz, PC*C*), 62.04 and 61.83 (2 × d, *J* = 6.2 Hz, 2 × POC), 45.82 (d, *J* = 15.6 Hz, PCC*C*), 42.55, 30.44 (d, *J* = 140.7 Hz, PC), 15.27 (d, *J* = 6.2 Hz, 2 × POC*C*) ppm; ^31^P NMR (81 MHz, CD_3_OD): *δ* = 31.11 ppm.

#### Diethyl 2-[2-[6-[bis(*tert*-butoxycarbonyl)amino]-9*H*-purin-9-yl]acetamido]-1-hydroxyethylphosphonate (**7g**, C_23_H_37_N_6_O_9_P)

According to the general procedure from 0.097 g diethyl 2-amino-1-hydroxyethylphosphonate (**10**, 0.492 mmol) and 0.290 g [6-[bis(*tert*-butoxycarbonyl)amino]-9*H*-purin-9-yl]acetic acid (**13g**, 0.492 mmol), the amide **7g** (0.142 g, 50% yield) was obtained as a colorless oil after purification on a silica gel column with chloroform–methanol mixtures (50:1, 20:1 v/v). IR (film): $\overline{V}$ = 3297, 3090, 2982, 2934, 2873, 1788, 1756, 1693, 1250, 1024 cm^−1^; ^1^H NMR (600 MHz, CDCl_3_): *δ* = 8.87 (s, 1H), 8.29 (s, 1H), 7.32 (t, *J* = 5.3 Hz, 1H, NH), 5.02 and 4.99 (AB, *J* = 16.3 Hz, 2H, C(O)C*H*_a_*H*_b_), 4.60 (t, *J* = 6.6 Hz, 1H, OH), 4.21–4.17 (m, 4H, 2 × POC*H*_2_CH_3_), 4.10–4.05 (m, 1H, PC*H*CH_2_), 3.91 (ddt, *J* = 14.1 Hz, *J* = 6.6 Hz, *J* = 3.7 Hz, 1H, PCC*H*_a_H_b_), 3.44–3.33 (m, 1H, PCCH_a_*H*_b_), 1.48 (s, 18 H, 6 × CH_3_), 1.36 and 1.35 (2 × t, *J* = 7.1 Hz, 2 × 3H, 2 × POCH_2_C*H*_3_) ppm; ^13^C NMR (151 MHz, CDCl_3_): *δ* = 166.60, 153.52, 152.08, 150.46, 150.25, 146.08, 128.39, 83.95, 66.61 (d, *J* = 163.0 Hz, PC), 63.23 and 63.08 (2 × d, *J* = 7.3 Hz, 2 × POC), 49.98, 41.60 (d, *J* = 5.8 Hz, PC*C*), 27.79, 16.43 (d, *J* = 5.5 Hz, 2 × POC*C*) ppm; ^31^P NMR (81 MHz, CDCl_3_): *δ* = 21.90 ppm.

#### Diethyl 3-[2-[6-(*tert*-butoxycarbonyl)amino]-9*H*-purin-9-yl]acetamido]-1-hydroxypropylphosphonate (**8j**, C_19_H_31_N_6_O_7_P × 2H_2_O)

According to the general procedure from 0.180 g diethyl 3-amino-1-hydroxypropylphosphonate (**11**, 0.852 mmol) and 0.335 g [6-[bis(*tert*-butoxycarbonyl)amino]-9*H*-purin-9-yl]acetic acid (**13g**, 0.852 mmol), the crude product **8g** contaminated with triethylamine hydrochloride (0.246 g, 49%) was obtained as a yellowish oil after purification on a silica gel column with chloroform–methanol mixtures (50:1, 20:1 v/v). Further purification with HPLC (water–methanol) gave pure **8j** (0.030 g, 7%) and a 7:3 mixture of **8j** and **8g** (0.125 g). Yellowish oil; IR (film): $\overline{V}$ = 3289, 3091, 2982, 2935, 1787, 1748, 1247, 1026 cm^−1^; ^1^H NMR (600 MHz, CDCl_3_): *δ* = 8.70 (s, 1H), 8.21 (brs, 1H), 8.12 (s, 1H), 7.64 (brt, *J* = 5.4 Hz, 1H), 4.95 (s, 2H, C(O)CH_2_), 4.61 (brs, 1H, OH), 4.21–4.12 (m, 4H, 2 × POC*H*_2_CH_3_), 4.03–3.98 (m, 1H), 3.65–3.60 (m, 1H), 3.46–3.41 (m, 1H), 2.09–1.95 (m, 1H), 1.93–1.84 (m, 1H), 1.58 (s, 9H, 3 × CH_3_), 1.33 and 1.32 (2 × t, *J* = 7.0 Hz, 2 × 3H, 2 × POCH_2_C*H*_3_) ppm;^13^C NMR (151 MHz, CDCl_3_): *δ* = 166.19, 153.41, 152.19, 150.57, 150.46, 145.55, 128.57, 84.00, 65.99 (d, *J* = 165.2 Hz, P*C*), 62.93 and 62.89 (2 × d, *J* = 6.3 Hz, 2 × POC), 46.40, 36.84 (d, *J* = 15.1 Hz, PCC*C*), 30.66 (PC*C*), 27.82, 16.49 (d, *J* = 5.5 Hz, 2 × POC*C*) ppm; ^31^P NMR (243 MHz, CDCl_3_): *δ* = 24.32 ppm.

#### Diethyl 3-[2-[6-(*tert*-butoxycarbonyl)amino]-9*H*-purin-9-yl]acetamido]-2-hydroxypropylphosphonate (**9j**, C_19_H_31_N_6_O_7_P × H_2_O)

According to the general procedure from 0.123 g diethyl 3-amino-2-hydroxypropylphosphonate (**12**, 0.582 mmol) and 0.229 g [6-[bis(*tert*-butoxycarbonyl)amino]-9*H*-purin-9-yl]acetic acid (**13g**, 0.582 mmol), the crude product **9g** contaminated with triethylamine hydrochloride (0.197 g, 57%) was obtained as a colorless oil after purification on a silica gel column with chloroform–methanol mixtures (100:1, 50:1 v/v). Further purification with HPLC (water–methanol) gave pure **9j** (0.019 g, 7%) and a 8:2 mixture of **9j** and **9g** (0.094 g). Colorless oil; IR (film): $\overline{V}$ = 3271, 30,881, 2992, 2955, 1782, 1760, 1234, 1023 cm^−1^; ^1^H NMR (200 MHz, CDCl_3_): *δ* = 8.70 (s, 1H), 8.12 (brs, 1H), 8.09 (s, 1H), 7.48 (brt, *J* = 6.2 Hz, 1H), 4.95 (s, 2H, C(O)CH_2_), 4.63 (brs, 1H, OH), 4.22–3.99 (m, 5H, 2 × POC*H*_2_CH_3_, PCC*H*), 3.62–3.46 (m, 1H), 3.37–3.24 (m, 1H), 2.10–1.88 (m, 2H), 1.56 (s, 9H, 3 × CH_3_), 1.30 (t, *J* = 7.0 Hz, 6H, 2 × POCH_2_C*H*_3_) ppm; ^13^C NMR (151 MHz, CDCl_3_): *δ* = 166.39, 152.92, 151.16, 149.98, 149.75, 143.30, 121.37, 82.30, 65.59 (d, *J* = 4.3 Hz, PC*C*), 62.18 and 62.15 (2 × d, *J* = 5.5 Hz, 2 × POC), 46.28, 46.04 (d, *J* = 18.4 Hz, PCC*C*), 30.90 (d, *J* = 140.2 Hz, PC), 28.14, 16.37 and 16.33 (2 × d, *J* = 4.6 Hz, 2 × POC*C*) ppm; ^31^P NMR (81 MHz, CDCl_3_): *δ* = 30.03 ppm.

#### Diethyl 2-[2-(2-amino-6-chloro-9*H*-purin-9-yl)acetamido]-1-hydroxyethylphosphonate (**7h**, C_13_H_20_ClN_6_O_5_P)

According to the general procedure from 0. 322 g diethyl 2-amino-1-hydroxyethylphosphonate (**10**, 1.63 mmol) and 0.372 g (2-amino-6-chloropurin-9-yl)acetic acid (**13h**, 1.63 mmol), the amide **7h** (0.278 g, 27%) was obtained as a white powder after purification on a silica gel column with chloroform–methanol mixtures (50:1, 20:1, 10:1 v/v). M.p.: 206–208 °C; IR (KBr): $\overline{V}$ = 3457, 3347, 3212, 3044, 2991, 2930, 1672, 1632, 1565, 1249, 1027, 705 cm^−1^; ^1^H NMR (200 MHz, CD_3_OD): *δ* = 8.10 (s, 1H), 4.94–4.88 (m, 2H, C(O)C*H*_a_*H*_b_), 4.30–4.13 (m, 4H, 2 × POC*H*_2_CH_3_), 4.08 (ddd, *J* = 9.1 Hz, *J* = 7.9 Hz, *J* = 3.7 Hz,1H, PC*H*CH_2_), 3.72 (ddd, *J* = 14.0 Hz, *J* = 7.3 Hz, *J* = 3.7 Hz, 1H, PCC*H*_a_H_b_), 3.48–3.37 (m, 1H, PCCH_a_*H*_b_), 1.38 (t, *J* = 7.0 Hz, 6H, 2 × POCH_2_C*H*_3_) ppm; ^13^C NMR (151 MHz, CD_3_OD): *δ* = 167.55, 160.31, 154.17, 150.18, 143.87, 123.23, 65.80 (d, *J* = 164.6 Hz, PC), 63.05 and 62.75 (2 × d, *J* = 7.3 Hz, 2 × POC), 41.17, 41.13 (d, *J* = 8.5 Hz, PC*C*), 15.40 and 15.73 (2 × d, *J* = 5.2 Hz, 2 × POC*C*) ppm; ^31^P NMR (81 MHz, CD_3_OD): *δ* = 23.67 ppm.

#### Diethyl 3-[2-(2-amino-6-chloro-9*H*-purin-9-yl)acetamido]-1-hydroxypropylphosphonate (**8h**, C_14_H_22_ClN_6_O_5_P × 0.75H_2_O)

According to the general procedure from 0. 366 g diethyl 3-amino-1-hydroxypropylphosphonate (**11**, 1.71 mmol) and 0.389 g (2-amino-6-chloropurin-9-yl)acetic acid (**13h**, 1.71 mmol), the amide **8h** (0.099 g, 14%) was obtained as a white powder after purification on a silica gel column with chloroform–methanol mixtures (50:1, 20:1, 10:1 v/v) and crystallization from methanol. M.p.: 183–184 °C; IR (KBr): $\overline{V}$ = 3401, 3278, 3213, 3089, 2980, 2935, 2864, 1690, 1631, 1223, 1031, 971 cm^−1^; ^1^H NMR (600 MHz, DMSO-*d*_*6*_): *δ* = 8.05 (s, 1H), 6.90 (brs, 2H, NH_2_) 5.50 (t, *J* = 7.0 Hz, 1H, OH), 4.73 (s, 2H, C(O)CH_2_), 4.11–3.94 (m, 4H, 2 × POC*H*_2_CH_3_), 3.87–3.71 (m, 1H, PC*H*), 3.45–3.25 (m, 2H, PCCC*H*_a_H_b_), 1.85–1.50 (m, 2H, PCCH_2_), 1.23 (t, *J* = 7.0 Hz, 6H, 2 × POCH_2_C*H*_3_) ppm; ^13^C NMR (151 MHz, DMSO-*d*_*6*_): *δ* = 166.56, 160.28, 154.88, 149.69, 144.56, 123.57, 64.46 (d, *J* = 164.6 Hz, P*C*), 62.28 and 61.94 (2 × d, *J* = 6.8 Hz, 2 × POC), 45.505, 36.08 (d, *J* = 17.1 Hz, PCC*C*), 31.60 (d, *J* = 2.6 Hz, PC*C*), 16.86 and 16.83 (2 × d, *J* = 5.0 Hz, 2 × POC*C*) ppm; ^31^P NMR (243 MHz, DMSO-*d*_*6*_): *δ* = 24.74 ppm.

#### Diethyl 3-[2-(2-amino-6-chloro-9*H*-purin-9-yl)acetamido]-2-hydroxypropylphosphonate (**9h**, C_14_H_22_ClN_6_O_5_P × 0.25H_2_O)

According to the general procedure from 0.485 g diethyl 3-amino-2-hydroxypropylphosphonate (**12**, 2.30 mmol) and 0.523 g (2-amino-6-chloropurin-9-yl)acetic acid (**13h**, 2.30 mmol), the amide **9h** (0.210 g, 22%) was obtained as a white powder after purification on a silica gel column with chloroform–methanol mixtures (50:1, 20:1, 10:1 v/v) and crystallization from an ethanol–diethyl ether mixture. M.p.: 163–164 °C; IR (KBr): $\overline{V}$ = 3399, 3280, 3245, 3091, 2981, 2936, 2907, 1690, 1614, 1224, 1031, 868 cm^−1^; ^1^H NMR (600 MHz, CD_3_OD): *δ* = 8.07 (s, 1H), 4.92 and 4.88 (AB, *J* = 16.6 Hz, 2H, C(O)CH_2_), 4.18–4.06 (m, 5H, 2 × POC*H*_2_CH_3_, PCC*H*), 3.42 (dd, *J* = 13.7 Hz, *J* = 4.8 Hz, 1H, PCCC*H*_a_H_b_), 3.31 (ddd, *J* = 13.7 Hz, *J* = 6.5 Hz, *J* = 1.0 Hz, 1H, PCCCH_a_*H*_b_), 2.11–1.98 (m, 2H, PCH_2_), 1.34 (t, *J* = 7.0 Hz, 6H, 2 × POCH_2_C*H*_3_) ppm;^13^C NMR (151 MHz, CD_3_OD): *δ* = 167.58, 160.31, 154.18, 150.20, 143.88, 123.28, 65.13 (d, *J* = 3.5 Hz, PC*C*), 62.08 and 61.83 (2 × d, *J* = 6.6 Hz, 2 × POC), 45.85 (d, *J* = 15.3 Hz, PCC*C*), 45.12, 30.62 (d, *J* = 140.9 Hz, PC), 15.25 (2 × d, *J* = 5.9 Hz, 2 × POC*C*) ppm; ^31^P NMR (243 MHz, CD_3_OD): *δ* = 29.94 ppm.

### Antiviral activity assays

The compounds were evaluated against different herpes viruses, including herpes simplex virus type 1 (HSV-1) strain KOS, thymidine kinase-deficient (TK^−^) HSV-1 KOS strain resistant to ACV (ACV^r^), herpes simplex virus type 2 (HSV-2) strain G, varicella-zoster virus (VZV) strain Oka, TK^−^ VZV strain 07-1, human cytomegalovirus (HCMV) strains AD-169 and Davis as well as vaccinia virus, adeno virus-2, human coronavirus, para-influenza-3 virus, reovirus-1, Sindbis virus, Coxsackie virus B4, Punta Toro virus, respiratory syncytial virus (RSV) and influenza A virus subtypes H1N1 (A/PR/8), H3N2 (A/HK/7/87) and influenza B virus (B/HK/5/72), were based on inhibition of virus-induced cytopathicity or plaque formation in human embryonic lung (HEL) fibroblasts, African green monkey kidney cells (Vero), human epithelial cervix carcinoma cells (HeLa) or Madin Darby canine kidney cells (MDCK). Confluent cell cultures in microtiter 96-well plates were inoculated with 100 CCID_50_ of virus (1 CCID_50_ being the virus dose to infect 50% of the cell cultures) or with 20 plaque forming units (PFU) and the cell cultures were incubated in the presence of varying concentrations of the test compounds. Viral cytopathicity or plaque formation (VZV) was recorded as soon as it reached completion in the control virus-infected cell cultures that were not treated with the test compounds. Antiviral activity was expressed as the EC_50_ or compound concentration required reducing virus-induced cytopathicity or viral plaque formation by 50%. Cytotoxicity of the test compounds was expressed as the minimum cytotoxic concentration (MCC) or the compound concentration that caused a microscopically detectable alteration of cell morphology.

### Cytostatic activity assays

All assays were performed in 96-well microtiter plates. To each well were added (5–7.5) × 10^4^ tumor cells and a given amount of the test compound. The cells were allowed to proliferate at 37 °C in a humidified CO_2_-controlled atmosphere. At the end of the incubation period, the cells were counted in a Coulter counter. The IC_50_ (50% inhibitory concentration) was defined as the concentration of the compound that inhibited cell proliferation by 50%.
